#  Recent Insights into the Involvement of Progranulin in Frontotemporal Dementia 

**DOI:** 10.2174/157015911798376361

**Published:** 2011-12

**Authors:** Li Sun, Jason L Eriksen

**Affiliations:** Department of Pharmacological and Pharmaceutical Sciences, University of Houston, Houston, TX, USA

**Keywords:** Frontotemporal lobar degeneration, progranulin, TDP-43, Alzheimer’s disease.

## Abstract

Progranulin is a widely expressed protein that is involved in the regulation of multiple biological processes, including embryogenesis, host defense, and wound repair. In the central nervous system, progranulin is constitutively expressed at modest levels in neurons and microglia, but shows dramatic microglial immunoreactivity in degenerative diseases that exhibit prominent neuroinflammation. In addition to the role that PGRN plays in the periphery, its expression is of critical importance in brain health, as demonstrated by recent discovery that progranulin haploinsufficiency results in familial frontotemporal lobar degeneration. Since progranulin deficiency was first described, there has been an intense ongoing effort to decipher the mysterious role that this protein plays in dementia. This review provides an update on our understanding of the possible neuronal function and discusses the challenging problems related to progranulin expression within genetics, cell biology, and neurodegeneration.

## INTRODUCTION

Progranulin (PGRN), alternatively described in the literature as pro-epithelin, granulin-epithelin precursor, PC-cell-derived growth factor and acrogranin [1, 2], is a highly conserved, widely expressed protein that fulfills a broad number of roles within a diverse set of tissues, regulating the rapidly dividing epithelial cells of the skin, gastrointestinal tract, leukocytes, and the reproductive system [3]. In humans, PGRN transcript is expressed from the *GRN* gene, situated on chromosome 17, consisting of 13 exons that encode 7.5 repeats of granulin peptide motifs, including paragranulin and granulins A-G. The full-length PGRN protein is 593 amino acids long, with a predicted molecular mass of 63.5kDa. 

As a secreted protein, PGRN fulfills numerous and complex regulatory roles in peripheral tissues. The peptide sequence contains an N-terminal signal directs the nascent protein into secretory transport; the secreted form of PGRN becomes glycosylated and has a molecular weight of approximately 90 kDa [4]. In the extracellular environment, PGRN can undergo cleavage by extracellular proteases including elastase, producing a series of 6kDa peptides [5, 6]. These peptides are rich in cysteine residues, and can undergo hairpin stacking to form the secondary structures thought to be necessary for biological activity [7]. 

There is evidence that PGRN and the granulin cleavage products may have opposing effects on cell cycling and growth [5, 8, 9], but the precise biochemical pathways, including the specific receptors for these proteins, and interplay between these different proteins on remains largely unknown. In peripheral tissues, PGRN cleavage is modulated by a balance of elastase and secretory leukocyte protease inhibitor (SLPI); SLPI can antagonize the elastase-mediated cleavage of PGRN [5, 6, 10]. The overall equilibrium between PGRN and the granulin products within the CNS, although currently not well-characterized, is thought to resemble the processes found within the periphery.

In the periphery, PGRN can promote tumorigenesis and can induce the expression of a series of inflammatory cytokines involved in inflammation and wound repair [1, 5, 6, 9]. PGRN gene expression is regulated by interleukin-1β and tumor necrosis factor-α, probably through a cytokine or hormone binding element in the promoter region [1, 11, 12]. Expression data in murine embryos supports the notion that PGRN is involved in embryogenesis and can promote embryonic development of the epidermis and spermatogenesis, and the formation of nervous system and blood vessels [13]. Both PGRN and the smaller granulins have the capability of promoting proliferation of epithelial cells, but appear to differ in their capacities for doing so [3]. In support of these findings, sex-linked behavioral abnormalities have been reported in PGRN-knockout mice [14, 15]. 

## PGRN IN THE CENTRAL NERVOUS SYSTEM

During embryonic development, PGRN promotes the onset of blastocyst cavitation [14-17]. It is universally expressed in the central nervous system during early neuronal development, and is required for neonatal steroid-induced sexual differentiation [13]. In rodents, PGRN is highly expressed in specific brain regions, including Purkinje cells, pyramidal and granule cells of the hippocampus, and the superficial lamina of cerebral cortex [3]. In neurologically normal patients, PGRN is similarly present in hippocampal pyramidal neurons, with a prominently strong signal localized to the in CA1, dentate fascia, and endplate/CA4 regions [1, 18-20]. 

The functional activities of PGRN are less well understood, but a similarly complex relationship is associated with the abilities of full length PGRN and the granulins in their effects on neuronal growth and survival [10]. *In vitro*, PGRN has been shown to promote growth of PC-12 neuronal cells from rat adrenal gland pheochro-mocytoma [3]. Studies of primary neurons show that full-length PGRN and the granulin fragment GRN-E support a prolonged period of neuronal survival in serum and neurotrophin-free media [10, 21]. When added to media, both full length PGRN and granulins can increase axonal outgrowth, although this effect appears to be most pronounced with the full-length PGRN. The ability of PGRN to promote neurite growth and neuronal survival appears to depend on cleavage into granulins. The addition of SLPI ablates these effects, similar to what is seen in tumorigenesis [5, 10], which implies a conserved PGRN function amongst these different tissues. 

It is not yet clear how granulins are produced in the central nervous system, but elastase and SLPI are thought to be produced by astrocytes and microglial cells; under these circumstances, a tightly-regulated network may be important to keep PGRN and granulins in balance [1, 22]. However, as our knowledge regarding the production of PGRN in the CNS is still rudimentary, it remains to be shown if SLPI works in a similar way in the central nervous system as it does within the periphery. 

## NEUROINFLAMMATION

PGRN expression is known to be significantly increased in association with neurodegenerative disease, with much of this data coming from a variety of unbiased expression array studies. The vast majority of these studies have implied that PGRN is upregulated within the CNS by neuroinflammation, whether this is due to infection, mechanical trauma, or associated with neurodegenerative processes. For instance, PGRN expression was found to be dramatically increased in mice infected with the Sindbis virus in the CNS [23]. 

Microglia are the predominant immunological defense mechanism in the central nervous system. It is know that when there are insults to the CNS, such as ischemia or hypoxia, that result in neuroinflammation [24], prolonged activation of microglia is able to cause secondary neuronal toxicity through the generation of proinflammatory cytokines and reactive oxygen species [25]. Full length PGRN may function as an immunomodulator to suppress excess microglial activation; PGRN-deficient hippocampal slices are highly vulnerable to oxygen and glucose deprivation, and show increased cell death in the presence of GM-CSF and LPS/IFN-gamma [9]. Conditional PGRN-knockout mice also exhibited exaggerated inflammatory tissue damage and delayed recovery when exposed to bacterial lipopolysaccharide, and showed showing an increased density of activated microglia during aging, when compared to wild-type mice [9].

PGRN appears to play a modulatory role in tissue damage within the CNS. Using a spinal cord injury mouse model, peak increase in PGRN mRNA and protein levels were observed at 7-14 days after injury, with activated microglia and macrophages being its main sources when evaluated by CD68 colocalization study [22]. The spatial and temporal expression of PGRN seems to be correlated with inflammation and recovery in this form of spinal cord neuronal injury. These finding parallel studies of a blunt-force traumatic brain injury model showing increased PGRN mRNA levels in hippocampus at twenty-four hours, a time when other neurotrophic factors began or have already decreased to normal levels [26].

Studies that have examined PGRN expression in neurodegenerative disorders, such as lysosomal storage diseases, amyotrophic lateral sclerosis (ALS), and Creutzfeldt-Jakob disease, show similar degrees of upregulation [1]. Compared with unaffected tissues in normal patients, in these disorders PGRN expression is prominently located in activated microglia [2, 18, 20]. In studies of Alzheimer’s disease, several reports have shown increased PGRN immunoreactivity is associated with amyloid plaques Fig. (**1**), dystrophic neurites, and microglia [27-29]. From these studies, it is not entirely clear whether PGRN is purely intracellular or is secreted and extracellularly associated with plaques; nevertheless, based upon previous observations, a substantial quantity of PGRN is likely to be contributed by activated microglia. 

While these studies add to our knowledge about the dynamic nature of PGRN expression in disease, more experiments are needed to establish the definitively establish to part that PGRN plays in modulating neuroinflammation. The appropriate expression of PGRN in a timely manner might be important in maintenance of brain functions. However, little is known about the mechanisms under which PGRN becomes upregulated in microglia under such conditions, and it remains unclear if this protein will be beneficial or detrimental once it becomes over-expressed in the nervous system. Although it is arguable that this increased expression of PGRN might be an effort to compensate and to rescue the neuronal functions as shown by its neurotrophic role in primary culture, it is possible that PGRN may exert a detrimental influence on the CNS, resulting in neuronal injury, especially if this protein is overexpressed on a long-term basis. 

## FRONTOTEMPORAL DEMENTIA

Patients diagnosed with frontotemporal dementia (FTD; alternatively identified as FTLD, frontemporal lobar degeneration) experience a prominent loss of language, significant changes of personality, and early alterations of social interactions and behaviors, characterized by the atrophy of the frontal and temporal lobes, with the relative sparing of the occipital and parietal lobes, sparing visual and spatial functions [30]. Familial forms of FTD account for the majority of all FTD cases, typically with an autosomal dominant inheritance pattern. Nevertheless, while many forms of FTLD display similar symptoms and are classified as early-onset dementias, this disorder can be subdivided into a set of disease entities with differences in clinical and pathological manifestations.

Clinically, FTLD patients may differ in their major symptoms, age of onset, and the course of the disease. Neuropathologically, FTLD can be divided into four subtypes according to its histology (Table **1**); this includes FTLD with tau deposits, FLTD with tau-negative, ubiquitin and TDP-43 positive inclusions (now called FTLD-TDP), and FTLD with neuronal intermediate filament inclusions (NIFID), and cases with no detectable inclusions [31]. The latter two subtypes have been combined into one group, FTLD-FUS, as FUS immunoreactivity is the uniform characteristic in these inclusions [32]. 

Four genes are currently associated with FTLD, including *MAPT* (microtubule-associated tau protein) on chromosome 17q21.32 [33-35], *VCP* (Valosin-containing protein) on chromosome 9p13.3 [36], *CHMP2B* (Chromatin modifying protein 2B ) on chromosome 3p11.2 [37], and *GRN* (PGRN) on chromosome 17q21.32 [20, 38, 39]. *MAPT* and GRN mutations are the most common forms of FTLD disease, with around 10-20% of familial FTLD patients with *MAPT* mutations, and a similar number of *GRN *mutation carriers [32]. *MAPT* mutations typically affect tau splicing at the mRNA level, or alter binding activity with microtubules but these mutations typically have little influence on total tau protein levels. In contrast, mutations in *GRN* play a predominant role in the development of FTLD pathology containing ubiquitinated, TDP-43-positive inclusions [39, 40]. The vast majority of *GRN* mutations that have been identified to the present cause a loss-of-function and decreased levels of PGRN expression through the introduction of a premature stop codon [41, 42].

The major neuropathology distinguishing FTLD associated with *GRN* mutations, from those without *GRN* mutations of PGRN is the presence of ubiquitin-positive lentiform neuronal intranuclear inclusions in the neocortex and striatum [2, 18, 39]. Other less significant characteristics include superficial laminar spongiosis, chronic neuronal degeneration, ubiquitin-immunoreactive neurites and ubiquitin-immunoreactive neuronal cytoplasmic inclusions. In these patients, ubiquitin-immunoreactive neurites are mostly seen in the striatum, and neuronal cytoplasmic inclusions in the hippocampus are usually present in a granular pattern. In contrast, no neuronal intranuclear inclusions are found in familial FTLD in patients lacking *GRN *mutations, along with less severe neocortical and striatal pathology. Patients diagnosed with FTLD-TDP demonstrate that PGRN immunoreactivity is most intensely found in activated microglia; PGRN so far has not been directly associated with ubiquitin-immunoreactive inclusions [1, 39]. 

### The Identification and Impact of GRN Mutations 

Since the first genetic linkage study identified the existence of *MAPT* gene mutations with familial FTLD [33-35], a significant amount of effort has been invested into the identification of novel genes associated with frontotemporal dementia, the second most common cause of senile dementia. Although several groups had reported an exclusive presence of tau-negative, ubiquitin-positive neuronal intranuclear inclusions in a subset of familial FTD patients without identifiable *MAPT* mutations, and others had narrowed potential candidate region to an area surrounding the *MAPT* [43-45], it was not until 2006 that *GRN* mutations were identified in familial FTLD *via *extensive candidate-gene sequencing [20, 39, 46]. Mutations in this gene were difficult to identify in part because it was 1.7 Mb proximal to the *MAPT* gene, a significant challenge for traditional genetic analysis. 

The seminal discovery of PGRN mutations in familial FTD resulted in a flurry of subsequent genetic screens and identification of novel familial mutations, expanding our understanding of the prevalence of *GRN* mutations and mechanisms of action in neurodegeneration. Currently, 68 PGRN mutations have been identified in 212 families (http://www.molgen.ua.ac.be/FTDMutations), including small insertions and deletions leading to a variety of frame-shift, nonsense, splice-site, and missense mutations (see reviews [41, 47, 48]); large-scale or complete deletion of *GRN* has been reported in several rare cases [47, 49]. These mutations have a common effect of reducing constitutive PGRN levels, but the underlying mechanisms through which this process occurs is likely to be different in some cases. Splice-site mutations may prevent the export of PGRN, subjecting PGRN to degradation [50]; in contrast, the most well-known of these is the haploinsufficiency mechanism; the introduction of premature stop codon results in nonsense-mediated RNA decay and a loss of 50 percent functional PGRN [20, 38, 48]. In addition to these mechanisms, less common mutations associated with a signal peptide motif appear to reduce PGRN expression by affecting the localization and secretion of the protein [19, 31, 51].

Based on the hypothesis that haploinsufficiency is a key contributor to neurodegeneration observed in FTLD-TDP, protein expression of PGRN in brain should, theoretically, always be reduced. However, in recent work [52] examining brain PGRN expression in FTLD-TDP patients with (PGRN+ FTLD-TDP) and without (PGRN- FTLD-TDP) *GRN* mutations, PGRN mRNA levels were surprisingly much higher in the most heavily affected brain region-frontal lobe in PGRN+ FTLD-TDP compared to those in PGRN- FTLD-TDP and neuropathologically-normal patients, while no significant differences were found in the other areas. Allele analysis indicated that the increased PGRN mRNA level came from the normal allele. Subsequent immuno-histochemistry staining suggested a prominent infiltration of microglia in frontal lobes, which might be the culprit serving increased PGRN mRNA levels in this condition. Protein levels remained lower for *GRN* mutation carriers in all four brain regions despite large variations among individuals in PGRN+ FTLD-TDP, potentially due to possible post-transcriptional modifications or failure of detection of full-length PGRN in ELISA. While the study points towards a role of microglial PGRN correlated with PGRN+ FTLD-TDP, the exact mechanism as to how this occurs is undetermined; additional patients will be needed to expand and confirm the distribution pattern of PGRN in FTLD-TDP associated with PGRN mutations. Comparative functional studies in different cell populations will be needed to address if there are two separate pools of PGRN in neurons and microglia cells within the CNS. 

### PGRN and TDP-43 in FTLD 

Widely expressed and predominantly nuclear, TDP-43 is a protein with 441 amino acids that is encoded by *TARDBP* gene on chromosome 1. It contains two RNA-recognition motifs and a C-terminal glycine-rich region that may mediate interactions with other proteins. It is a pleiotropic protein implicated in the regulation of gene expression including transcription, RNA splicing and transport, and translation [53, 54] and in maintenance of cell survival [55]. Apart from its role in gene regulation, other functions of this protein were largely unknown. However, in late 2006, Aria [56] and Neumann [57] independently reported that the heavily ubiquitinated inclusions in the brains of FTLD-TDP and amyloid lateral sclerosis (ALS) patients were predominantly composed of abnormally hyperphosphorylated and ubiquitinated TDP-43 protein, indicating a possible common pathogenic mechanism. As *GRN* mutation carriers invariably develop TDP-43 positive inclusions, these studies propelled the development of studies to assess the relationship between PGRN expression and TDP-43 patholog [20, 38]. Since the initial publication of these findings, a large number of *GRN* mutation variants have been identified in FTLD-TDP families across the world, strongly supporting the relationship between PGRN expression and the development TDP-43 pathology [32]. 

It is interesting to note that reduced levels of PGRN are also highly associated with the development of TDP-43-positive pathology in Alzheimer’s disease in the form of hippocampal sclerosis [58, 59]. Since the majority of GRN mutations have been reported to create null alleles, resulting in reduced protein levels, it is suspected that the protein might be a potential neurotrophic factor whose loss of function may justify the underlying mechanisms of neurodegeneration in FTLD-TDP or other related diseases [1]. In fact, little immunoreactivity of PGRN was detectable in degenerating neurons, consistent with the consequence *via *haploinsufficiency mechanism [39].

Although the initial discovery of aggregated TDP-43 inclusions represented a tremendous advance in the field, these studies did not show if TDP-43 pathology was merely a byproduct of other pathogenic progress, or was a casual factor resulting in neurodegeneration. The following year after TDP-43 aggregates were described in ALS and FTLD-TDP, dominant mutations in the *TARDBP* gene were reported by several groups as a primary cause of ALS in a variety of populations [42, 60-62]. These studies collectively provided evidence that the aberrant form of TDP-43 can directly trigger neurodegeneration. To date, 29 TARBP missense mutations have been reported excluding those benign gene variants, with all but one localized in the C-terminal glycine-rich region [60, 61, 63]. 

Cluster mutations in the highly conserved c-terminal region of TDP-43 identified in ALS may affect its transportation through the nuclear pore by interfering with the normal protein-protein interactions [60, 61]. All *TARDBP* mutations exhibit an autosomal dominant pattern of inheritance, and are almost exclusively seen in ALS. Most recently, two patients with FTLD plus motor neuron diseases (FTLD-MND) bearing a *TARDBP* mutation were also reported [64], illustrating that the pathogenic mutations, although rare, are not limited to ALS. While the studies of mutations in *TARDBP* have provided a number of powerful observations, it was unclear whether TDP-43 mutations caused neuronal death through a loss of normal function or through a gain-of-toxic function. 

Studies using different models suggest both pathways may potentially be important. Normally, TDP-43 shuttles between the nuclear and the cytoplasm exerting its biological functions in a transcription-dependent manner. The C-terminus is closely related to the solubility of the protein [55]; mutant forms of TDP-43 bearing the Q331K are more prone to aggregation [65]. Using *in vitro* cell models, TDP-43 Q331K or M337V missense mutations were shown to be associated with embryonic growth defects *via *pro-apoptotic effect in a chicken embryo model [61], suggesting a potential toxic gain of function. In support of the loss of function hypothesis, *Drosphila* lacking TDBH, the TDP-43 homolog protein, exhibits motor defects and abnormal synaptic terminals, a phenotype that can be rescued by human TDP-43 or TDBH expression [66]. 

While these observations provide significant insights into the genetics and cell biology of TDP-43, the relationship of TDP-43 and PGRN is still not well-characterized. In cell studies, PGRN knockdown leads to a caspase-3 dependent degradation of TDP-43, generating two fragments with the apparent molecular weight of 25kDa and 35kDa, respectively [67]. These findings suggest a possible mechanism of PGRN insufficiency and neurodegeneration through the production of generating toxic fragments of TDP-43. Indeed, the 25kDa fragment is able to induce cell toxicity *via *formation of insoluble, hyperphosphorylated and ubiquitin-positive inclusions in the cytosol of H4 cells, consistent with what is seen in FTLD-TDP inclusions [68]. Unfortunately, no data was shown if PGRN overexpression could rescue the degradation observed in these cell models [67].

In addition to TDP-43, FUS/TLS, another DNA/RNA binding protein, was recently identified as a causative gene in a subset of ALS [69, 70]; increased FUS immuno-reactivity in FTLD subtypes constitute a new group that combines the neuropathology with undefined histology and with intermediate neurofilament inclusions [32]. No FUS mutations or variability have been identified in FTLD as far. Considering similar roles of FUS and TDP-43 involved in gene regulation, it might be reasonable to suspect that dysfunction of RNA metabolism, suggestive of a loss-of-function model, might be the common pathway leading to neurodegeneration, regardless of the initial etiology or genetic heterogeneity [54]. 

Attempts to model the association between PGRN and TDP-43 using transgenic mice have so far only partially recapitulated the neurodegenerative phenotype observed in humans, suggesting that there are some key underlying functional differences between mouse models and in FTLD-TDP patients that are not currently understood. None of the PGRN mouse lines that have been developed so far, either as partial or complete knockouts, show the severe and progressive neurodegenerative phenotype that is associated with FTLD-TDP patients. The transgenic mouse models, however, do appear to show some signs of dysfunction. One of the earliest characterized PGRN KO mouse lines displayed alterations in neuroinflammation, focal neuronal loss, and the development of ubiquitin-positive lipofuscin during aging, but without clear changes in TDP-43 localization or distribution [71]. In contrast, another mouse model of PGRN was reported to have altered distribution of TDP-43 from nucleus to cytoplasm in hippocampal and thalamus; there was some disruption in learning and memory tests, although without clear evidence for cerebral atrophy [9, 72]. 

### Modifiers of PGRN Expression

Although mutations in *GRN* are correlated with the development of several well-characterized subtypes of FTLD neuropathology, genetic studies of families with *GRN* mutations have shown that there is substantial variability of clinical phenotypes in the aspect of age of onset, disease duration, severity and detailed manifestations [48]. The clinical heterogeneity suggests that there might be additional gene and environmental factors that can modify the progression of the disease. Indeed, several single nucleotide polymorphisms (SNPs) have been reported to be associated with sporadic FTLD without causal *GRN* mutations. The neuronal nitric oxide synthase (NOS) C276T polymorphism is associated with a substantially greater risk of developing FTLD (OR=1.96, 95% CI: 1.11-3.47) in a small Italian cohort (n=71 for cases) [73]. In another study, (n=222), the authors found that the endothelial NOS G893T polymorphism might increase the risk of FTLD by 65% [74], and the GG genotype of rs4859146 SNP in defective in cullin neddylation 1 (*DCN-1*)-domain containing 1 (*DCUN1D1*), involved in protein degradation, increased the risk more than 3-fold [75]. While the findings above suggested that the pathways of oxidative stress and protein degradation might be involved in FTLD, the results should be treated with caution as these studies used only clinically diagnosed FTLD patients in a single population; the possibility of false association cannot be excluded unless confirmed by larger studies in different populations. The neuropathology of FTLD patients and controls was also not available and could represent a mixture of different subtypes.

In a series of pathologically-confirmed FTLD patients without *GRN* mutations, Rademakers and colleagues demonstrated that a common genetic variant (rs5848) in a binding-site for microRNA miR-659, located in the 3'-untranslated region (UTR) of PGRN, was a major susceptibility factor for FTLD-TDP [42]. TT genotype of rs5848 increased the risk to develop FTLD-TDP by around 3 fold compared with CC genotype (95% CI: 1.50-6.73; n=339 for cases and n=934 for controls); PGRN levels were significantly reduced in TT homozygotes [42]. miR-659 appears to confer an increased risk for FTLD-TDP through an inhibition of PGRN translation, generating an effect resembling the biochemistry and pathology observed in PGRN- null mutations. This association was independently confirmed by another group showing that the rs5848 TT genotype resulted in a decrease in *GRN* mRNA levels over the CC genotype. A recent follow-up study examined the serum of patients with the rs5848 genotype [76], and showed that patients in the CC subgroup displayed the highest level of serum PGRN, CT group had intermediate values, and the TT group had the lowest level. Although this study utilized a small cohort of subjects, it appears to confirm the biological association between variations in R5848 and PGRN levels. It should be noted that two groups reported that they were unable to replicate the effect of the rs5848 polymorphism [77, 78]. The discrepancy might be due to several possible reasons such as a relatively weak or modifiable effect of the T allele, a smaller percentage or specific differences in the neuropathology of FTLD-TDP cohorts. 

In addition to miR-659, a genome-wide association study identified three single nucleotide polymorphisms at chromosome 7p21 [79], involving 545 cases of neuropathologically-confirmed FTLD-TDP patients. The corres-ponding region includes *TMEM106B*, a gene encoding an unrecognized transmembrane protein with 274 amino acids, with a preferable expression in the frontal lobe. The mRNA levels are significantly higher in FTLD-TDP patients compared to controls and in FTLD-TDP with *GRN* mutations, compared to non-carriers. Analysis of the heterozygotes on SNP site rs1990622 showed that the presence of PGRN mutation remains associated with increased TMEM106B expression, indicating that the *TMEM106B* genotype is not only a genetic risk factor for overall FTLD, but may probably confer additional risk for PGRN mutation carriers. In follow-up studies, an association analysis demonstrated a highly significant correlation between *TMEM106B* and PGRN plasma levels and mRNA expression, with greater statistical significance in patients with FTLD with *GRN *mutations [80]; these results suggested that *TMEM106B* expression modulates the development of FTLD through its interactions with PGRN. This protein appears to interact with PGRN to influence its activity in FTLD-TDP, although the function of TMEM106B remains poorly understood.

### Prognostic Biomarkers for FTLD-TDP

As most of the *GRN* variants associated with FTLD-TDP are null mutations, additional efforts have been directed towards developing methods, other than genetic analysis, for early diagnosis of the disease, especially in patients with atypical symptoms. Since the first report that mRNA levels in blood cells were significantly reduced in PGRN mutation carriers by approximately 35% [81], PGRN is decreased in plasma [82], serum [83], and cerebral spinal fluid in FTLD patients bearing the PGRN mutations but remains unchanged in those without PGRN mutations or normal patients. Finch and colleagues [84] reported that the plasma levels of full-length PGRN were significantly decreased in both symptomatic and asymptomatic PGRN mutation carriers, compared to relatives without PGRN mutations. Although this observation needs to be confirmed in additional FTLD families, these findings offer the promise that plasma PGRN levels might be used as a screening tool to predict the PGRN mutation status especially in those asymptomatic members of the family, which is valuable for evaluation of possible preventive care. 

While PGRN is a secreted protein that can be readily detected in plasma, it is somewhat surprising that TDP-43, a predominantly nuclear protein, is also detectable in plasma with ELISA [85]; raised levels of TDP-43 were detected in 46% of FTLD patients, 22% of Alzheimer’s disease and 8% of controls. In studies of ALS patients in a Japanese population, elevated levels of TDP-43 in the cerebral spinal fluid suggested this diagnostic may be of value at the early stages of disease [86]. Consequently, plasma levels of TDP-43 protein may also be of value in diagnosing FTLD patients with TDP-43 inclusions. Nevertheless, it is important to not out that diagnosis of the disease is purely correlative at this point. In order to judge the utility of this approach, a larger cohort of patients with neuropathologically confirmed diagnoses will be needed to verify if TDP-43 levels in blood plasma indeed appropriately correlate with TDP-43 pathology in the brain. 

## THERAPEUTIC APPROACHES TO PGRN HAPLOINSUFFICIENCY

Although the role that PGRN plays in the nervous system is as yet largely undefined, two groups, complimentary approaches of genetic and functional screening recently independently reported Sortilin (*SORT1*) as a high affinity neuronal receptor for PGRN [87, 88]. Sortilin is a member of the cellular Vps10 key type I receptor, which is expressed largely on neurons, and this receptor binds a number of different ligands, including the neuropeptide neurotensin and pro-NGF. 

In studies of the functional interaction between sortilin and PGRN, mice lacking the sortilin receptor had elevated levels of serum and brain PGRN, indicating that sortilin functions an important endogenous regulator of PGRN levels; however, overexpression of PGRN did not affect sortilin levels. As shown in Fig. (**2**), follow-up studies showed that PGRN was targeted to the endosomal-lysosomal pathway in neurons, suggesting that this protein plays a potentially important role in the degradation of proteins, but the precise function of this protein within the lysosome as yet remains undefined [87]. Although much work remains to be done to define the role of PGRN within the nervous system, these recent discoveries suggest that medications that targeting the sortilin receptor may be of significant utility in treating FTLD-TDP.

## CONCLUDING REMARKS

Despite many studies that have demonstrated the impact of *GRN* haploinsufficiency on brain health, our under-standing of the functional role of PGRN and its relationship to neurodegeneration is still nascent. Functional studies suggest that this protein has pleiotropic activities, fulling roles as both a full-length protein and in the form of protein fragments. In studies of peripheral tissues, PGRN exerts an impact on development, inflammation, tissue repair and oncogenesis. There appear to be strong parallels between the periphery and CNS in the regulation of this protein by inflammation within the microglial population, but the func-tion of PGRN within neurons, and its relationship to TDP-43 expression, remains much less understood. Although it has been commonly believed that a simple reduction in PGRN levels will result in neurodegeneration, this assumption has been challenged by recent work showing high levels of PGRN expression in FTLD-TDP patient population, and raises the question of whether PGRN produced by the two subpopulations of neurons and microglia fulfills an identical function in both cell types. If the role of PGRN is identical between neurons and microglia, this would argue that any increase in PGRN would be sufficient to rescue PGRN haploinsufficiency, whether this comes from a neuronal or microglia population. However, in most diseases it appears likely, although has not been entirely proven, that most of the PGRN is provided by activated microglia caused by neuronal inflammatory events. Future studies to dissect the differences in function in the neuronal and microglial populations, both in health and disease, and the relationship of this expression to TDP-43, will prove to be very informative in elucidating the function of this protein and its role in neurodegeneration.

## Figures and Tables

**Fig. (1):**
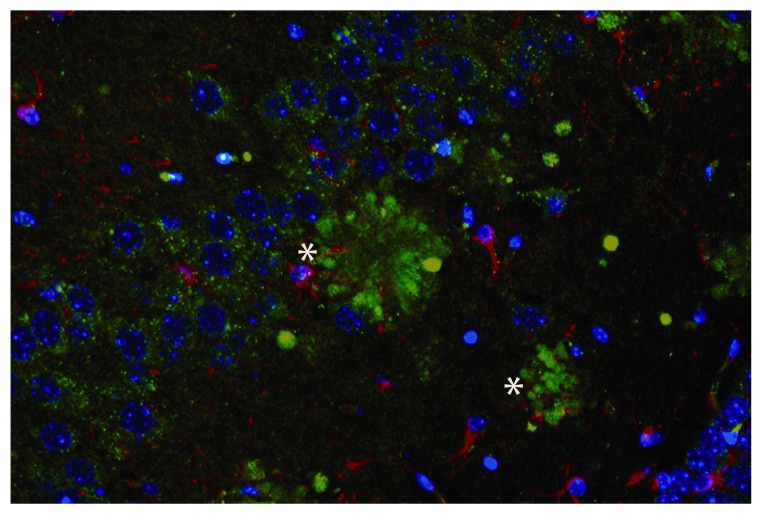
Immunofluorescence image from the hippocampus of a 15 month-old Tg2576 mouse model of Alzheimer’s disease, showing PGRN staining. A majority of amyloid plaques (indicated here by *) display intensely immunoreactivity for PGRN. Key: Green, PGRN; Red, glial fibrillary acidic protein; Blue , DAPI.

**Fig. (2):**
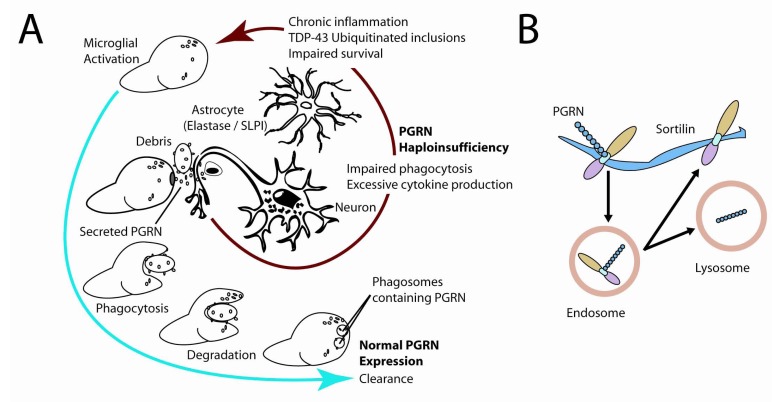
Depiction of potential functional roles of PGRN in the CNS **A**. During neuroinflammation, such as is associated with infections or neurodegenerative diseases, current data suggests that the majority of endogenous PGRN is produced and secreted by microglia into the surrounding extracellular space. Existing work suggests that in instances of haploinsufficiency there is an exaggerated neuroinflammatory response, potentially leading toin the neurodegenerative phenotype observed in FTLD-TDP **B**. The activity of PGRN is likely mediated *via* the endosomal-lysosomal pathway in part through its interactions with sortilin, a VPS10p receptor. PGRN binds tightly to this receptor, and the complex undergoes translocation into the endosomal-lysosomal compartment. Sortilin is recycled to the plasma membrane, whereas PGRN is transported and accumulates within the lysosome.

**Table 1.** 2010 Recommendations by Mackenzie [89] for Classification of FTLD Subtypes. The Subtype Indicates A Characteristic Pattern of Underlying Pathology, Rather than Clinical Syndrome Displayed by the Patient

Major Class	Subtype	Associated Gene
**FLTD-tau**	Pick’s disease	*MAPT*
	Corticobasal degeneration	
	Progressive supranuclear palsy	
	Argyrophilic grain disease	
	Multiple system tauopathy with dementia	
	Neurofibrillary tangle predominant dementia	
	White matter tauopathy with globular glial inclusions	
	Unclassifiable	
**FTLD-TDP**	Types 1-4	*GRN*
	Unclassifiable	9p (*TARDBP*)
**FTLD-UPS**	FTD-3	*CHMP2B*
**FTLD-FUS**	Atypical frontotemporal lobar degeneration with ubiquitinated inclusions	*FUS*
	Neuronal intermediate filament inclusion disease	
	Basophilic inclusion body disease	
**FTLD-ni**	No inclusions	
